# An Oscillating-Flow Microfluidic PCR Method for Rapid and Flexible Detection of Periodontal Pathogens

**DOI:** 10.3390/s26072126

**Published:** 2026-03-29

**Authors:** Zhenqing Li, Yueqing Wang, Jing Yang, Bo Yang, Yuan Zeng, Shinichi Sekine, Yoshinori Yamaguchi

**Affiliations:** 1 Engineering Research Center of Optical Instrument and System, Ministry of Education, Shanghai Key Lab of Modern Optical System, University of Shanghai for Science and Technology, No. 516 JunGong Road, Shanghai 200093, China; zhenqingli@usst.edu.cn (Z.L.);; 2 Faculty of Engineering, Anhui Sanlian University, Hefei 230601, China; yangjing@mail.slu.edu.cn; 3 College of Medical Imaging, Shanghai University of Medicine & Health Sciences, Shanghai 201318, China; 4 Department of Preventive Dentistry, Graduate School of Dentistry, Osaka University, Osaka 565-0871, Japan; 5 Comprehensive Research Organization, Waseda University, Tokyo 162-0041, Japan

**Keywords:** microfluidic chip, polymerase chain reaction, periodontal pathogens, *Porphyromonas gingivalis*, *Treponema denticola*

## Abstract

Polymerase chain reaction (PCR) is widely regarded as the gold standard for nucleic acid analysis; however, conventional thermal cycling limits its applicability in rapid and compact analytical systems. Here, we report an oscillating-flow microfluidic PCR method that enables rapid and flexible amplification by repeatedly shuttling the reaction mixture between two fixed-temperature zones. Unlike continuous-flow PCR, the proposed approach decouples PCR cycle number from microchannel geometry, allowing programmable cycling while reducing chip footprint. To enhance analytical reliability, polymer-assisted surface passivation using polyvinylpyrrolidone was employed to suppress nonspecific adsorption in polydimethylsiloxane (PDMS) microchannels, significantly improving amplification efficiency. Using *Porphyromonas gingivalis* and *Treponema denticola* as representative periodontal pathogens, 35-cycle amplification was completed within 20 min with reliable product yield. The proposed method advances oscillating-flow PCR toward a robust analytical strategy for rapid pathogen detection and related microfluidic nucleic acid analysis.

## 1. Introduction

Polymerase chain reaction (PCR) remains the benchmark technique for nucleic acid analysis owing to its high specificity, sensitivity, and reproducibility, and has been extensively applied in clinical diagnostics [1,2], food safety [3,4], and biological research [5,6]. Nevertheless, conventional benchtop PCR systems rely on block-based thermal cycling with limited heating and cooling rates, typically requiring 50–60 min to complete a standard amplification protocol. This intrinsic limitation restricts their applicability in time-sensitive and point-of-care analytical scenarios.

Microfluidic PCR technologies have been widely investigated as an effective strategy to accelerate thermal cycling by reducing thermal mass and enhancing heat transfer efficiency. Existing microfluidic PCR platforms can be broadly categorized into static-chamber PCR chips [7,8,9] and continuous-flow PCR (CF-PCR) [10,11] systems. Static-chamber PCR chips essentially miniaturize conventional PCR, but remain constrained by the need for repeated temperature cycling of the same reaction chamber, resulting in limited improvements in total assay time [12,13]. Continuous-flow PCR, in contrast, achieves rapid amplification by transporting the reaction mixture through spatially separated temperature zones, thereby converting time-dependent thermal cycling into spatial temperature control [14,15].

Despite the demonstrated advantages of CF-PCR, several intrinsic limitations hinder its broader analytical application. First, the number of PCR cycles is geometrically fixed once the microchannel layout is defined, limiting flexibility for assay optimization. Second, the requirement for long serpentine microchannels increases device footprint and sample dispersion, which is unfavorable for compact system integration. Third, precise flow regulation often relies on external syringe pumps, increasing system complexity and reducing portability.

To address these challenges, oscillating-flow PCR has emerged as an alternative strategy that combines the rapid heat transfer characteristics of CF-PCR with the flexibility of programmable cycle numbers [16]. In oscillating PCR systems, the reaction mixture is repeatedly shuttled between discrete temperature zones, allowing precise control over cycle number while significantly reducing chip area [17]. Representative studies have demonstrated the feasibility of oscillating-flow PCR for amplifying generic DNA targets [18,19]. However, these proof-of-concept demonstrations have not systematically addressed several critical analytical challenges. Specifically, two key technical bottlenecks still persist: (i) nonspecific biomolecule adsorption onto microchannel surfaces compromises amplification efficiency and reproducibility; and (ii) integrated analytical workflows enabling real-time monitoring and product validation remain underdeveloped. Consequently, translating oscillating-flow PCR from a structural concept toward a robust analytical method for clinical diagnostics—particularly for detecting clinically relevant pathogens—remains an ongoing challenge.

In this study, we present a compact oscillating-flow microfluidic PCR system tailored for the rapid detection of periodontal pathogens. Two key anaerobic bacteria associated with periodontitis—*Porphyromonas gingivalis* (*P.g*) and *Treponema denticola* (*T.d*)—were selected as representative analytical targets [20,21,22]. To enhance analytical reliability, we employed polyvinylpyrrolidone (PVP)-assisted surface passivation to effectively mitigate nonspecific biomolecule adsorption within the PDMS microchannels. The system further integrates programmable oscillatory flow with an optimized thermal configuration, enabling flexible-cycle PCR amplification with rapid heat exchange. Under these conditions, 35 amplification cycles are reliably completed within 20 min. During operation, fluorescence signals are continuously acquired from the microchannel, and the resulting intensity profiles are analyzed to assess the generation of characteristic amplification curves. This real-time fluorescence monitoring supports robust evaluation of nucleic acid amplification performance and analytical consistency. Therefore, this work advances oscillating-flow PCR from a conceptual microfluidic architecture toward a practical, application-oriented analytical platform for rapid pathogen detection.

## 2. Materials and Methods

### 2.1. Materials

DNA Ladder at 100 bp (Cat: M1200), 5× Tris-borate-EDTA (TBE) buffer (Cat: T1050) and 2× SYBR Green PCR Mastermix were bought from Solarbio (Cat:SR1110, Beijing, China). Hydroxyethylcellulose (HEC) (Cat: 434981-250G, 1300 k) (SigmaAldrich, Shanghai, China) was purchased from SigmaAldrich (Shanghai, China). Speed STAR HS DNA Polymerase was from Takara (Cat: RR070A, Beijing, China). Additionally, 0.085 mM Polyvinylpyrrolidone was obtained from Aladdin (Cat: P274371-100g, Shanghai, China). The primers were synthesized by Sangon (Shanghai, China).

### 2.2. Construction of Oscillating Flow PCR Microfluidic System

**Figure 1:**
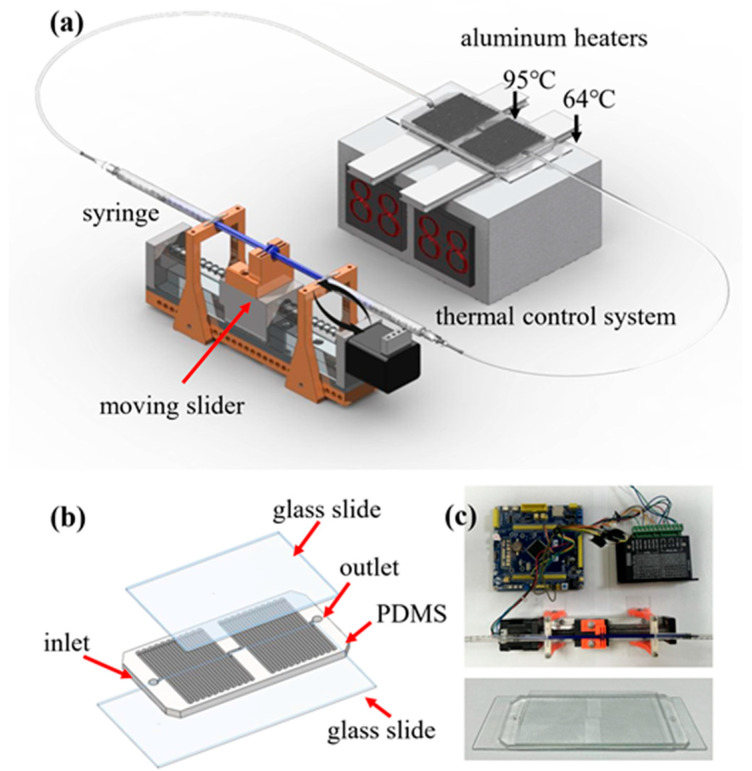
Schematic illustration of the oscillating-flow PCR microfluidic system and the fabricated chip. (**a**) Overall configuration of the oscillating PCR platform, including a syringe pump that drives the PCR solution back and forth through the microchannel, a moving stepping motor for controlled oscillatory motion, and a thermal control module composed of aluminum heaters that maintain two temperature zones (95 °C for denaturation and 64 °C for annealing/extension). (**b**) Structural layout of the microfluidic PCR chip consisting of a PDMS microchannel layer sandwiched between two glass slides, with designated inlet and outlet ports for sample loading. (**c**) Photographs of the assembled experimental setup, including the control electronics and the fabricated microfluidic chip used in the oscillating PCR experiments.

The self-built oscillating flow PCR microfluidic system (Figure 1a) consists of a constant temperature control system, stepping motor, syringe, microcontroller, microfluidic chip and power supply. The brackets supporting each part were manufactured by 3D printing technology. The device was connected to the power supply, and the temperature required for the PCR reaction was adjusted using the constant temperature control system. After the temperatures of the two aluminum heating blocks reached the desired temperatures, the conditions required for the PCR reaction were set on the computer software. The oscillating flow PCR chip was fixed on two aluminum blocks, and the stepping motor drove the syringe to repeated flow of the reaction liquid in the channel between the two temperature zones. The main part of the microfluidic system is shown in Figure 1c. The amplified PCR products were determined in a self-built capillary electrophoresis (CE) system.

To further clarify temperature uniformity across the microchannel during oscillatory operation, both structural design and thermal configuration were considered. The PDMS microchannel was tightly bonded to a 170 μm glass substrate and directly placed on independently controlled aluminum heating blocks, forming spatially stable denaturation and annealing zones. The high thermal conductivity of the glass slide promotes lateral heat spreading, minimizing temperature gradients across the channel width. Moreover, the shallow channel height (100 μm) ensures rapid thermal equilibration between the fluid and channel wall.

### 2.3. Construction of Oscillating Flow PCR Microfluidic Chip

As shown in Figure 1b, the microchannels of the microfluidic chip are distributed on the left and right sides of the chip, corresponding to the high temperature area and the low temperature area, respectively. The length, width and height of the meandering microchannel are 0.88 m × 1000 μm × 100 μm. The entire microfluidic chip is equipped with two interfaces, which are connected to two syringes that control the oscillation and inlet and outlet of the reaction solution. To reduce high-temperature evaporation, 0.17 mm-thick glass slide is used for double-sided bonding. The purpose of one side is to form a microfluidic channel, and the other side is to avoid evaporation of PCR solution from the microfluidic chip, because PDMS is a porous material. The microchannel dimensions were determined by jointly considering heat transfer efficiency, oscillation-induced residence time, and PCR reaction requirements. While the denaturation and annealing zones are defined by dedicated heating modules, the extension zone relies on controlled fluid residence time, achieved by adjusting oscillation velocity, allowing sufficient extension without additional heating and reducing system complexity.

The microchannel dimensions (0.88 m in total length, 1000 μm in width, and 100 μm in height) were determined through comprehensive consideration of thermal, fluidic, and biochemical requirements. The shallow channel height (100 μm) minimizes thermal mass and enables rapid heat transfer between the fluid and channel wall, ensuring efficient thermal cycling. The relatively wide channel (1000 μm) reduces hydraulic resistance and facilitates stable bidirectional oscillation driven by the stepping motor without excessive backpressure. The total channel length provides sufficient residence time within each temperature zone during oscillation to allow complete denaturation, annealing, and extension. Importantly, unlike continuous-flow PCR, the cycle number is programmable and not geometrically constrained by channel length.

### 2.4. Preparation of the Photomask

The pattern and the photomask of the microfluidic chip was designed by AutoCAD 2023 and fabricated by lithography. SU-8 2050 photoresist was spin-coated (1750 r/min, 30 s) on a silicon wafer after it was processed by plasma machine, and a photoresist coating with a thickness of 100 μm was obtained. Next, it was baked at 65 °C for 5 min and 95 °C for 17 min to remove organic solvents. Subsequently, the mask was aligned with the silicon wafer and subjected to UV exposure to form a 3D structure of the chip pattern. Then, the SU-8 was further cured by baking at 65 °C for 4 min and 95 °C for 9 min. Next, the silicon wafer was subsequently developed in a developer solution to remove the unexposed areas. Finally, it was baked at 180 °C for 15 min to solidify the mold.

### 2.5. Fabrication of the Oscillating Flow PDMS Microfluidic Chip

First, the silicon wafer was fumigated with 1H, 1H, 2H, 2H-perfluorooctyltrichlorosilane (PFTS) for 30 min in a fume hood to form a highly hydrophobic perfluorochemical film on the surface of the silicon wafer. Next, the prepolymer and cross-linking agent were mixed ihn a ratio of 10:1, stirred until milky white, and degassed in a vacuum environment for 1 h to remove bubbles. Then, the PDMS mixture was poured onto the treated silicon wafer, vacuum-degassed for 1 h, and then placed in an 80 °C oven for curing for 1 h. After curing, the PDMS was peeled off the silicon wafer, cut to appropriate size, and exit holes punched using a 0.7 mm punch. Finally, the PDMS sheet and the glass sheet were rapidly bonded after plasma treatment to form a closed microfluidic channel.

### 2.6. Capillary Electrophoresis System

Determination of the PCR products was performed on a self-built capillary electrophoresis system [23], which consists of a high-voltage power supply (MODEL 610E, TREK, Medina, OH, USA), an upright fluorescence microscope (BX51, Olympus, Tokyo, Japan), a photomultiplier tube R928 (Hamamatsu Photonics, Hamamatsu, Japan), and a fused-silica capillary (ID/OD = 75/365 μm, Polymicro Technologies, Phoenix, AZ, USA). The fused-silica capillary had a total length of 15 cm, with an effective length of 8 cm. The working principle of the CE system is as follows: The DNA–dye complex was driven into the capillary filled with hydroxyethylcellulose (HEC) under an applied DC voltage of 1500 V. As the complex passed through the detection window, it was excited by light corresponding to the wavelength of the fluorescent dye and emitted fluorescence, which was collected by the PMT. The fluorescence signal was subsequently converted into digital data using a PCI-6024E data acquisition card (National Instruments, Austin, TX, USA). Both the high-voltage power supply and data acquisition were controlled using self-developed software.

## 3. Results and Discussion

### 3.1. Evaluation of the Temperature Distribution

The temperature distribution within the microchannel is a critical factor determining the successful amplification of target DNA on the chip. To evaluate the thermal characteristics of the system, a glass slide was first placed above the aluminum heating blocks, and the preset temperatures of the heaters were set to 96 °C and 65 °C, corresponding to the denaturation and annealing/extension zones, respectively. The surface temperature distribution was initially assessed using an infrared (IR) thermal camera (Testo 865, Testo, Inc., Shanghai, China) at an ambient temperature of approximately 25 °C. It demonstrated that the temperature across the heater surface was spatially uniform. The temperature measured on the top surface of the glass slide was approximately 1 °C lower than the heater setpoint, indicating predictable heat loss across the glass interface and the necessity of slightly elevating the preset heater temperature to achieve the desired reaction temperature within the microchannel.

**Figure 2:**
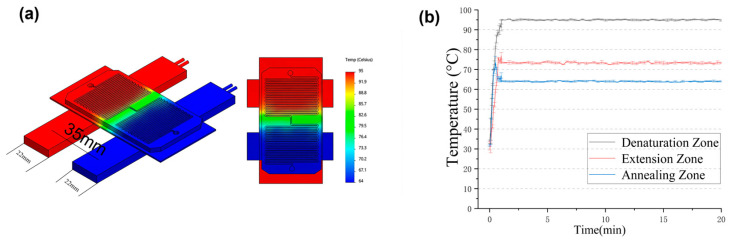
(**a**) Numerical results and (**b**) real-time temperature profiles of the denaturation, annealing, and extension zones during oscillating-flow PCR microfluidic chip.

We performed a thermal simulation analysis of the microfluidic PCR chip using COMSOL Multiphysics ®6.1 (Figure 2a). The relevant physical parameters are listed in Table S1 in the Supporting Information. The 3D model shows the two aluminum heating blocks (red and blue) and the microfluidic chip (green) placed on top. The color map on the right represents the temperature distribution across the system, ranging from cool (blue, ~64 °C) to hot (red, ~95 °C). The simulation results indicate a spatially uniform temperature field within each of the three distinct thermal zones: the denaturation zone (red), the extension zone (green), and the annealing zone (blue). The temperature gradient between the zones is clearly visible, and the transition regions are well defined. To further characterize the thermal behavior under dynamic operating conditions, the temperature of the glass slide surface was monitored using a TCM-M207 temperature sensor (EasyShining Technology, Chengdu, China). As shown in Figure 2b, real-time temperature profiles were recorded in the denaturation, annealing, and extension zones during oscillating-flow operation. Temperature data were collected every 5 s during the heating phase and every 15 s after the temperature stabilized, with each measurement repeated three times. These results indicate that the oscillatory motion does not disrupt the established thermal field and that the fluid experiences highly consistent temperature conditions upon each traversal of a given zone. Consequently, temperature uniformity in the proposed system is achieved dynamically, ensuring reproducible thermal exposure during successive PCR cycles.

### 3.2. The Effect of PVP on the PCR Efficiency for the Microfluidic Chip

The PCR reagent usually contains biological macromolecules (e.g., DNA template), primers, Taq DNA polymerase and dNTPs, etc. The inside surface of the microchannel may absorb these components. It will not only reduce the PCR efficiency, but may also inhibit the amplification. Thus, it is necessary to reduce its adsorption by passivating the inside surface. As a water-soluble polymer, PVP is often used as an anti-adhesion agent in biological experiments, such as preventing the adsorption of Taq DNA polymerase in PCR microfluidic chips. To investigate the effect of PVP on the PCR efficiency, we took *P.g* (197 bp) as an example, and performed its amplification in the microfluidic chip. For comparison, we also performed the PCR in a T100 thermal cycler (Bio-Rad, Hercules, CA, USA). The thermal cycling consisted of 95 °C for 2 min, and 35 cycles of denaturation at 95 °C for 10 s and annealing/extension at 64 °C for 20 s. Finally, the PCR products were stored at 4 °C. The primers and the composition of the PCR solution for traditional PCR and oscillating flow PCR are listed in Table 1 and Table 2, respectively.

**Figure 3:**
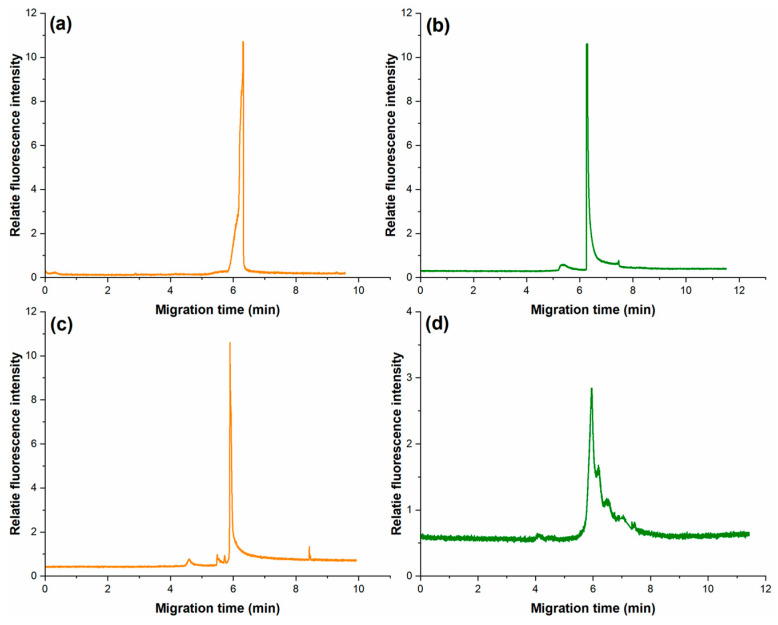
The electropherogram PCR products of *P.g* amplified by T100 thermal cycler (**a**) containing PVP and (**b**) without PVP, and in the microfluidic system (**c**) containing PVP and (**d**) without PVP. Electrophoretic conditions: 0.5% HEC (1300 k), 100 V/cm of electric field strength, total length 15 cm and effective length 7 cm of the capillary.

We separately amplified *P.g* in the T100 thermal cycler and the oscillating flow PCR chip within and without PVP. Data in Figure 3a,b show that PVP did not have an obvious effect for the traditional PCR. PVP does not affect the migration time of PCR products detected in CE, but it can affect the volume of the PCR products in the oscillating flow PCR microfluidic system (Figure 3c,d). The amount of product reduces non-specific amplification. This is possibly because the vinyl group in PVP has a significant passivation effect on the PDMS microchannel surface, mainly by preventing the non-specific adsorption of Taq DNA polymerase. Moreover, it indicates the good solubility and biocompatibility of PVP.

To assess the reproducibility of PVP-enhanced amplification, experiments were performed in triplicate under identical conditions. The migration times of the 197 bp *P.g* amplicon showed high consistency, with relative standard deviations (RSD) of 0.8% and 1.1% for the T100 thermal cycler and microfluidic chip, respectively. Fluorescence intensity measurements from three independent runs exhibited coefficients of variation (CV) of 9.2% for the conventional system and 12.4% for the microfluidic system, indicating acceptable reproducibility given the manual operation involved in chip-based assays.

We also evaluated other common antifouling strategies for microfluidic PCR, including Tween-20 and BSA, by replacing PVP in the PCR mixture with 1 mg/mL BSA or Tween-20. During experiments, 6 µL of Tween-20 caused flow difficulties and blockages, so we diluted it by 50%. *P.g* was amplified under BSA and Tween-20 conditions using both a conventional thermal cycler and the oscillating-flow PCR chip, with three repeats per condition. With BSA, additional peaks appeared in capillary electrophoresis (CE) detection for both systems (Figure S1a,b). This may be due to BSA’s large molecular weight and heterogeneity, which can interfere with fluorescent dye, causing non-specific signals. With Tween-20, target amplification succeeded in both systems. No impurity peaks were observed in conventional PCR (Figure S1c,d), but extra peaks appeared in the oscillating-flow system. This difference likely arises from the dynamic conditions in the oscillating-flow chip, where continuous fluid motion may alter interfacial properties, flow stability, and mixing, potentially leading to non-specific amplification during CE analysis.

### 3.3. The Effect of PCR Cycle Number

**Figure 4:**
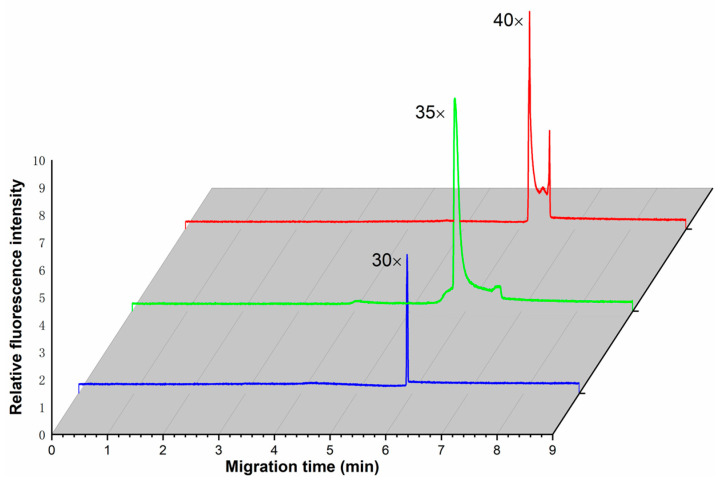
The electropherogram of PCR products running in the microfluidic chip with 30, 35 and 40 PCR cycle number. The electrophoretic conditions were as those in Figure 3.

To determine the optimal cycle number for the oscillating-flow PCR system, we amplified the 197 bp target gene of *P.g* using 30, 35, and 40 cycles under otherwise identical conditions. Figure 4 shows the electropherograms of the PCR products obtained at each cycle number. The relative fluorescence intensity increased markedly when the cycle number was raised from 30 to 35, but showed only marginal further increase at 40 cycles. Moreover, non-specific amplification products became evident at 40 cycles, as indicated by additional peaks in the electropherogram. To assess reproducibility, each cycle condition was performed in triplicate using freshly prepared PCR mixtures. The fluorescence intensities of the 197 bp product exhibited coefficients of variation (CV) of 11.3%, 8.7%, and 14.2% for 30, 35, and 40 cycles, respectively. The higher variability observed at 40 cycles is likely attributable to the onset of non-specific amplification, which introduces competition for reagents and increases well-to-well variation.

Based on these results, 35 cycles were selected as the optimal condition for subsequent experiments, offering the best combination of high product yield, minimal non-specific amplification, and acceptable reproducibility (CV = 8.7%). To monitor amplification progress in real time, we additionally evaluated the fluorescence intensity of the PCR mixture at a fixed detection point within the microchannel using a monochrome camera (MV-XG170GM-T, MindVision, Shenzhen, China) with 480 nm laser excitation. The *P.g* template was mixed with 2× SYBR Green PCR Mastermix prior to introduction into the chip. Fluorescence intensity, quantified from grayscale values of captured images (see Figure S1 in Supporting Information), increased progressively over time, and reached a plateau at approximately 20 min, consistent with the completion of 35 amplification cycles.

### 3.4. Time-Dependent Fluorescence Evolution in the Microfluidic Chip

**Figure 5:**
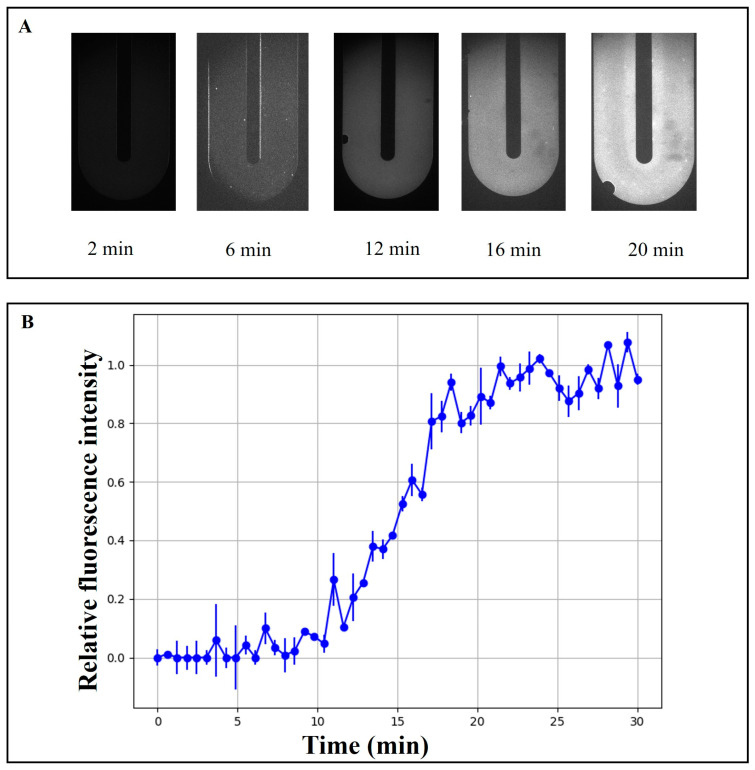
(**A**) The fluorescence intensity varied with reaction time. (**B**) Real-time amplification curve assessment.

We evaluated the fluorescence intensity of the PCR mixture at a specific point on the microfluidic chip using the above monochrome camera, with excitation of the PCR product by a 480 nm laser. The *P.g* template was mixed with 2× SYBR Green PCR Mastermix, and the resulting mixture was introduced into the microfluidic chip for amplification. The volume of the PCR product was calculated from the grayscale values of the captured images. Figure 5 illustrates the time-dependent fluorescence evolution in the microfluidic chip. As shown in Figure 5A, fluorescence images were captured at a fixed region of interest within the microchannel at different time points (2, 6, 12, 16, and 20 min). The fluorescence signal was initially weak and gradually increased over time, indicating the progressive accumulation of fluorescent products within the reaction chamber. A noticeable enhancement in signal intensity was observed after approximately 12–16 min, suggesting the onset of rapid reaction kinetics.

To quantitatively characterize this dynamic process, the grayscale fluorescence intensity was extracted from the selected region and normalized to its maximum value. As presented in Figure 5B, the normalized fluorescence intensity exhibited a sigmoidal growth trend over time. The signal remained near baseline during the early stage (0–10 min), followed by a rapid increase between 12 and 18 min, and eventually reached a plateau after approximately 20 min. Error bars represent the standard deviation from repeated measurements, demonstrating good reproducibility of the fluorescence detection system.

### 3.5. Amplification of Periodontal Pathogens in the Oscillating Flow PCR Microfluidic System

**Figure 6:**
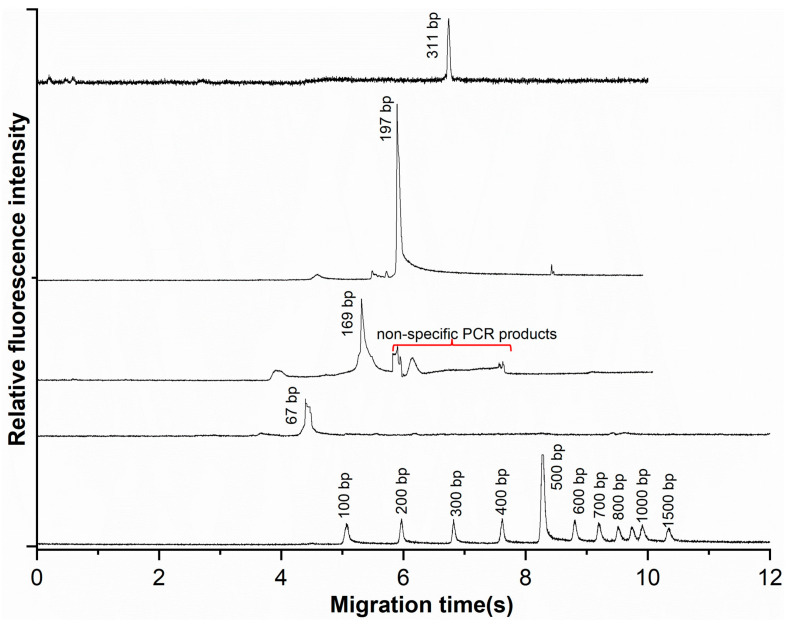
The electropherogram of the PCR products and 100 bp DNA ladder. The electrophoretic conditions were as those in Figure 3. The red line points to the non-specific PCR products.

To evaluate the system for clinically relevant targets, we amplified four fragments from Porphyromonas gingivalis (*P.g*, 67 bp, 169 bp, 197 bp) and Treponema denticola (*T.d*, 311 bp). PCR mixtures containing 0.085 mM PVP and 0.1% Tween-20 were oscillated between 95 °C and 64 °C zones for 35 cycles (∼20 min). Products were analyzed by CE (100 V/cm, 0.5% HEC). As shown in Figure 6, distinct peaks for all four targets were observed, with migration times of 4.67 min (*P.g*-67 bp), 5.61 min (*P.g*-169 bp), 5.91 min (*P.g*-197 bp), and 6.74 min (*T.d*-311 bp). Amplicon sizes were confirmed by linear fitting against a 100 bp ladder (R = 0.998). A minor non-specific peak was also noted (red line in Figure 6).

Reproducibility was assessed in triplicate experiments. Migration times showed high consistency across all targets (RSD < 2%), with *P.g*-67 bp, *P.g*-169 bp, *P.g*-197 bp, and *T.d*-311 bp exhibiting migration times of 4.67 ± 0.09 min (RSD 1.9%), 5.61 ± 0.06 min (RSD 1.1%), 5.91 ± 0.05 min (RSD 0.8%), and 6.74 ± 0.08 min (RSD 1.2%), respectively. Fluorescence intensities exhibited moderate variability, with coefficients of variation (CV) of 18.4%, 12.0%, 9.0%, and 11.8% for the four targets in ascending size order. The higher CV and lower intensity of *P.g*-67 bp likely reflect suboptimal primer design (high GC content, Tm mismatch), underscoring the importance of primer optimization.

These results demonstrate analytical feasibility under controlled laboratory conditions using purified DNA. However, this represents a proof-of-concept validation rather than clinical validation. Clinical specimens (plaque, saliva) contain PCR inhibitors and complex microbial backgrounds not replicated here. Further validation with clinical samples and comparison with gold-standard methods (e.g., qPCR) is needed to establish diagnostic utility.

### 3.6. Comparison with Reported PCR Microfluidic Methods

To clarify the analytical advancement of the proposed oscillating-flow PCR system, its performance was compared with representative microfluidic PCR platforms reported in recent literature (Table 3). Static-chamber PCR chips typically require 20–45 min for 35–40 cycles due to repeated heating and cooling of the reaction chamber [12,13]. Continuous-flow PCR systems can reduce amplification time to 10–25 min [24,25,26]. However, they require long microchannels to define cycle numbers, resulting in large chip footprints and limited flexibility.

In contrast, the present oscillating-flow PCR system decouples PCR cycle number from channel geometry by repeatedly shuttling the reaction mixture between two fixed-temperature zones. This design allows programmable cycle numbers without increasing channel length, thereby reducing chip area while preserving rapid amplification. Compared with previously reported oscillating PCR devices, the present work further incorporates polymer-assisted surface passivation using PVP, which significantly improves amplification efficiency in PDMS microchannels by suppressing nonspecific adsorption of polymerase and nucleic acids. Analytically, the system enables completion of 35 PCR cycles within approximately 20 min, comparable to or faster than most reported CF-PCR platforms [19], while offering improved flexibility and reduced system complexity. Moreover, amplification of representative periodontal pathogens was demonstrated under controlled laboratory conditions, indicating the feasibility of applying the proposed system to clinically relevant DNA targets. It should be noted that the current experiments serve primarily as a proof-of-concept validation. Further studies involving more complex biological samples and comparison with established clinical detection methods will be necessary to fully assess the clinical applicability of this approach.

## 4. Conclusions

In summary, we have developed and analytically evaluated an oscillating-flow microfluidic PCR system for rapid pathogen detection. By combining spatial temperature control with programmable oscillatory flow, the system overcomes the fixed-cycle limitation and large footprint associated with conventional continuous-flow PCR chips. Polymer-assisted surface passivation using PVP effectively mitigated nonspecific adsorption within PDMS microchannels, resulting in improved amplification efficiency without affecting conventional benchtop PCR. Using *P. gingivalis* and *T. denticola* as representative targets, the system achieved reliable amplification within 20 min for 35 PCR cycles, with good reproducibility demonstrated across triplicate experiments. Compared with existing microfluidic PCR approaches, the proposed method offers a balanced combination of speed, flexibility, and compactness, advancing oscillating-flow PCR toward practical analytical applications. We acknowledge that a systematic evaluation of the detection limit and dilution series was not performed in this study; these aspects will be addressed in future work to further establish the analytical performance of the system. Future work will also focus on parallelization and high-throughput integration to enhance its applicability in clinical and point-of-care diagnostics.

## Figures and Tables

**Table 1:** Primers of the periodontal pathogens.

Target	Sequence (5′ to 3′)	Amplicon (bp)
*P.g*	Fw: GCGCTCAACGTTCAGCC Rw: CACGAATTCCGCCTGC	67
*P.g*	Fw: TGACCTTAAGCCTGTAG Rw: TCATCTCGTGTCCGATCACT	169
*P.g*	Fw: TGTAGATGACTGATGGTGAAAACC Rw: ACGTCATCCCCACCTTCCTC	197
*T.d*	Fw: AAGGCGGTAGAGCCGCTCA	311
	Rw: AGCCGCTGTCGAAAAGCCCA	

**Table 2:** Composition of the PCR solution employed for traditional PCR and oscillating microfluidic PCR system.

Reagents	Volume (µL)	Volume (µL)
*P.g*	1	1
Forward primer (10 mM)	1	1
Reverse primer (10 mM)	1	1
dNTPs	4	4
Speed STAR HS DNA Polymerase	0.25	0.25
10× Fast Buffer I	5	5
PVP	0	4
Tween-20	0	2
ddH_2_O	37.75	31.75

**Table 3:** Qualitative comparison of representative microfluidic PCR methods reported in the literature.

PCR Method Category	Thermal Cycling Strategy	PCR Cycle Flexibility	Typical Amplification Time (35 Cycles)	Chip Footprint	Main Analytical Limitations	Representative References
Static-chamber PCR microfluidic chip	Time-dependent thermal cycling of a fixed chamber	High	35–45 min	Small to medium	Limited speed due to low thermal ramping rate	Refs. [12,13]
Continuous-flow PCR (CF-PCR)	Spatially separated temperature zones with unidirectional flow	Fixed (defined by channel geometry)	10–25 min	Large (long serpentine microchannels)	Fixed cycle number; large footprint; limited flexibility	Refs. [24,26]
Reported oscillating-flow PCR systems	Oscillatory flow between discrete temperature zones	Moderate	~20–30 min	Medium	Limited analytical optimization; surface adsorption effects often not addressed	Refs. [17,27]
This work	Oscillatory flow between two fixed-temperature zones combined with polymer-assisted surface passivation	High (fully programmable)	~20 min	Compact	—	This study

## Data Availability

The data presented in this study are available on request from the corresponding author due to institutional and ethical considerations, as the dataset contains information that could potentially compromise participant privacy.

## Supplementary Materials

The following supporting information can be downloaded at: https://www.mdpi.com/article/10.3390/s26072126/s1, Figure S1: Capillary electrophoresis of PCR products amplified under different antifouling conditions: PCR solution containing (a) BSA in the T100 thermal cycler and (b) the oscillating-flow microfluidic chip; PCR solution containing Tween-20 in (c) the T100 thermal cycler and (d) the oscillating-flow microfluidic chip; Table S1: Physical property of Water, Polydimethylsiloxane (PDMS), and Glass.
