# Supplementary material for: An Oscillating-Flow Microfluidic PCR Method for Rapid and Flexible Detection of Periodontal Pathogens
Source: Sensors (Basel). 2026 Mar 29;26(7):2126. doi: 10.3390/s26072126 (PMC13075334; doi:10.3390/s26072126)
Supplement: Supplementary file 1 [file sensors-26-02126-s001.zip › sensors-4199036-supplementary.pdf]

Supporting Information

An Oscillating-Flow Microfluidic PCR Method for Rapid and Flexible Detection of Periodontal Pathogens

Zhenqing Li [1], Yueqing Wang [1], Bo Yang [1], Jing Yang [2], Yuan Zeng [3], Shinichi Sekine [4], Yoshinori Yamaguchi [5,]

[1] Engineering Research Center of Optical Instrument and System, Ministry of Education, Shanghai Key Lab of Modern Optical System, University of Shanghai for Science and Technology, No. 516 JunGong Road, Shanghai 200093, China

[2] Faculty of Engineering, Anhui Sanlian University, Hefei, 230000, China

[3] College of Medical Imaging, Shanghai University of Medicine &Health Sciences, 201318, Shanghai, China.

[4] Department of Preventive Dentistry, Graduate School of Dentistry, Osaka University, Osaka, Japan.

[5] Comprehensive Research Organization, Waseda University, Tokyo 162-0041, Japan.

Corresponding Author
* Yoshinori Yamaguchi, yoshi.yamaguchi@ap.eng.osaka-u.ac.jp.

Table S1. Physical property of Water,Polydimethylsiloxane(PDMS),and Glass

Fluid	Density (kg/m3)	Specific heat (j/kg*k)	Thermal conductivity (w/m*k)	Viscosity (kg/m*s)
Water	998.2	4182	0.6	0.001003
Polydimethylsiloxane	970	1400	0.15	--
Glass	2500	750	1.0	--

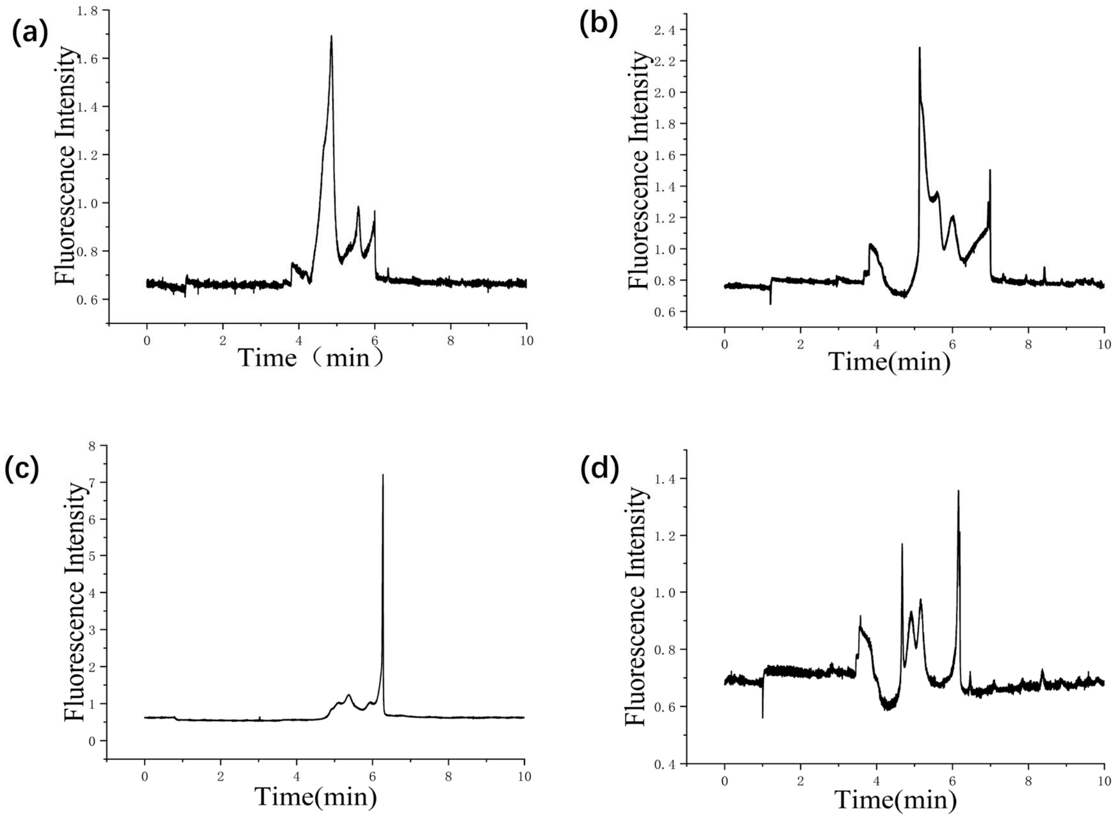

Figure S1. Capillary electrophoresis of PCR products amplified under different antifouling conditions: PCR solution containing (a) BSA in the T100 thermal cycler and (b) the oscillating-flow microfluidic chip; PCR solution containing Tween-20 in (c) the T100 thermal cycler and (d) the oscillating-flow microfluidic chip.
